# Formulation of Piperine–Chitosan-Coated Liposomes: Characterization and In Vitro Cytotoxic Evaluation

**DOI:** 10.3390/molecules26113281

**Published:** 2021-05-29

**Authors:** Syed Sarim Imam, Sultan Alshehri, Mohammad A. Altamimi, Afzal Hussain, Wajhul Qamar, Sadaf Jamal Gilani, Ameeduzzafar Zafar, Nabil K. Alruwaili, Saleh Alanazi, Bjad K. Almutairy

**Affiliations:** 1 Department of Pharmaceutics, College of Pharmacy, King Saud University, Riyadh 11451, Saudi Arabia; simam@ksu.edu.sa (S.S.I.); maltamimi@ksu.edu.sa (M.A.A.); afzal.pharma@gmail.com (A.H.); Sa4270909@yahoo.com (S.A.); 2 Central Laboratory, Research Center, College of Pharmacy, King Saud University, Riyadh 11451, Saudi Arabia; wqidris@ksu.edu.sa; 3 Department of Basic Health Sciences, Preparatory Year, Princess Nourahbint Adbulrahman University, Riyadh 11671, Saudi Arabia; gilanisadaf@gmail.com; 4 Department of Pharmaceutics, College of Pharmacy, Jouf University, Aljouf Region, Sakaka 72341, Saudi Arabia; zzafarpharmacian@gmail.com (A.Z.); nkalruwaili@ju.edu.sa (N.K.A.); 5 Department of Pharmaceutics, College of Pharmacy, Prince Sattam Bin Abdulaziz University, Alkharj 11942, Saudi Arabia; b.almutairy@psau.edu.sa

**Keywords:** piperine, liposomes, chitosan, breast cancer, MCF-7, mucoadhesive

## Abstract

The present research work is designed to prepare and evaluate piperine liposomes and piperine–chitosan-coated liposomes for oral delivery. Piperine (PPN) is a water-insoluble bioactive compound used for different diseases. The prepared formulations were evaluated for physicochemical study, mucoadhesive study, permeation study and in vitro cytotoxic study using the MCF7 breast cancer cell line. Piperine-loaded liposomes (PLF) were prepared by the thin-film evaporation method. The selected liposomes were coated with chitosan (PLFC) by electrostatic deposition to enhance the mucoadhesive property and in vitro therapeutic efficacy. Based on the findings of the study, the prepared PPN liposomes (PLF3) and chitosan coated PPN liposomes (PLF3C1) showed a nanometric size range of 165.7 ± 7.4 to 243.4 ± 7.5, a narrow polydispersity index (>0.3) and zeta potential (−7.1 to 29.8 mV). The average encapsulation efficiency was found to be between 60 and 80% for all prepared formulations. The drug release and permeation study profile showed biphasic release behavior and enhanced PPN permeation. The in vitro antioxidant study results showed a comparable antioxidant activity with pure PPN. The anticancer study depicted that the cell viability assay of tested PLF3C2 has significantly (*p* < 0.001)) reduced the IC_50_ when compared with pure PPN. The study revealed that oral chitosan-coated liposomes are a promising delivery system for the PPN and can increase the therapeutic efficacy against the breast cancer cell line.

## 1. Introduction

Piperine (PPN) is the major bioactive compound found in black pepper. It has been widely used as a food supplement but also has many therapeutic properties in different diseases. Its therapeutic efficacy has been reported against diseases such as inflammation, obesity, CNS diseases and neurodegeneration protection [1,2]. The use of PPN has also been reported in different types of cancer. It has shown activity against B16F-10 melanoma cells [3] and also in B (α)-p (benzopyrene)-induced experimental lung cancer [4]. In recent studies, PPN has also shown activity against breast cancer by targeting cancer stem cell renewal properties [5]. Breast cancer cannot be cured only by surgery, and there is a need for adjunct chemotherapy [6]. Chemotherapeutic agents can cause secondary malignancies [7] and severe side effects due to their inability to discriminate between healthy and proliferating cancer cells [8]. Breast cancer cells become resistant to chemotherapy due to the emergence of variants that express P-glycoprotein [9]. Thus, using natural bioactive compounds with an anticancer property is a useful alternative to breast cancer chemotherapy treatment. These natural compounds mostly have lesser side effects and variant effects against all breast cancer cell subtypes (luminal A, luminal B, basal-like and HER2-enriched) [6]. PPN has been reported in the treatment of different cancers. Its activity has also been reported in breast cancer. It acts by blocking the cell cycle at the G2/M-phase breast cancer cell line (4T1 cells) by increasing the phosphorylation level of cyclin-dependent kinase-1 and kinase-2 [10,11]. In another study, Jain et al. reported that PIP increased the generation of the intracellular ROS level in HeLa and MCF-7, which arrest the cell cycle at the G2/M phase with decreased cell viability [12]. It also inhibits the spouting process of rat aorta in angiogenesis induced by breast cancer cells [13]. PPN is a hydrophobic compound with limited solubility (~40 µg/mL) [1,14]. Thus, the therapeutic use of PPN is limited. Therefore, there is a need for novel formulations to improve the dissolution and solubility of PPN. A number of nanosized delivery systems have been reported for the anticancer activity of PPN, such as piperine with paclitaxel liposomes [15], apamycin and piperine polymeric nanoparticles [16], piperine micelles [17], PIP hydroxyapatite nanoparticles [18], curcumin and piperine emulsomes [19] and protopanaxadiol-piperine nanoparticles [20]. They have reported enhanced activity from individual PIP-loaded nanoformulations, as well as combined delivery with PIP with other anticancer drugs.

The encapsulation of drug into vesicular delivery systems has been widely used to enhance the solubility, stability, therapeutic efficacy and bioavailability of poorly soluble drugs [21,22,23]. Liposomes are widely used as a delivery system for hydrophilic and hydrophobic drugs [24,25]. Liposomes contain a lipid bilayer that is similar to the cell membrane. They are composed of phospholipids, cholesterol and surfactant. Liposomes are considered nontoxic, biodegradable and modulate the release properties [26]. However, liposomes can show instability when given orally due to chemical and enzymatic degradation. Therefore, chitosan (C) coating is performed over the surface of the liposomes to modify the surface, leading to better liposomal characteristics. Chitosan is a natural polysaccharide that has gained attention among researchers as a functional material. It is nontoxic, biodegradable, biocompatible and has a mucoadhesive property [27]. It forms a surface coat over the liposomes by the electrostatic deposition method, creating a positively charged chitosan–liposome complex [28]. Thus, the interaction with the negatively charged cell membrane helps widen the tight junction between epithelial cells, allowing for the transport of macromolecules [24,27,29,30].

Therefore, the present study was designed to prepare PPN-loaded liposomes and chitosan-coated liposomes to compare physicochemical parameters. The selected formulations were further characterized for mucoadhesive study, permeation study and antioxidant activity. Finally, the selected chitosan-coated PPN liposomes were evaluated for cytotoxic effects in the MF7 breast cancer cell line.

## 2. Results and Discussion

### 2.1. Formulation Optimization

Piperine-loaded liposomes were prepared with the composition shown in Table 1. From the preliminary study, the composition of the liposomes was optimized based on size and encapsulation efficiency. The selected four formulations were prepared with phosphatidylcholine, cholesterol and sodium cholate. The formulation was prepared with chloroform and methanol as an organic solvent blend to dissolve the used excipients. The ratio was finalized to prepare the thin lipid film based on the solubility of PPN, cholesterol, phosphatidylcholine and sodium cholate in the chloroform and methanol (1:2) blend. Finally, the formulations were hydrated with phosphate-buffered saline to prepare the PPN liposomes. The prepared formulations PLF2 and PLF3 were selected to be coated with chitosan to enhance the mucoadhesive property. The two concentrations of chitosan (0.2% and 0.4% *w*/*v*) were taken to coat the prepared liposomes and further characterize.

### 2.2. Vesicle Size

**Figure 1:**
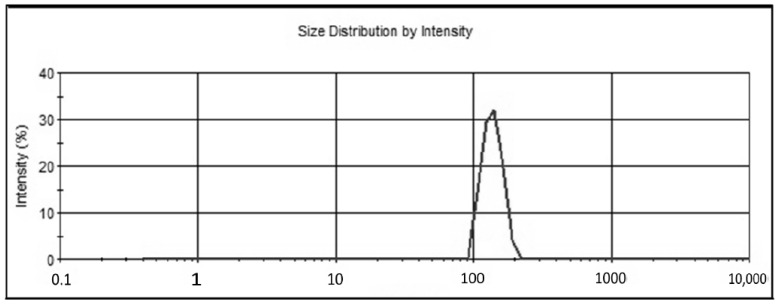
Vesicle size image of chitosan-coated PPN liposomes (PL3C1).

The prepared PPN liposomes showed a vesicle size in the range of 165.7 ± 7.4 to 213.1 ± 6.3 nm (Table 2). The variation in size was found due to the different ratios of cholesterol and surfactant. The formulation PLF4 showed the smallest size among the prepared liposomes, and the formulation PLF1 showed the largest size. The increase in lipid content led to an increase in size. The two formulations PLF2 and PLF3 were selected to be coated with chitosan (0.2% and 0.4%) based on their size and EE. Furthermore, results showed a marked enhancement in size due to the chitosan coating on the lipid vesicles. The formulation PLF2 has a size of 178.1 ± 4.5 nm, and after chitosan coating (PLF2C1), the size increased to 243.4 ± 7.5 nm (Figure 1). The uncoated and coated liposomes showed a narrow size distribution. The values were found below 0.3, which confirms the narrow size distribution and size homogeneity. The surface charge is the key factor for the stability of the formulations. It indicates the degree of electrostatic repulsion between the particles. The high zeta potential value (±30 mV) is considered to be stable. The highly charged particles do not aggregate due to electric repulsion. The low zeta potential value leads to the attraction of particles due to the van der Waals attractive forces and may lead to the formation of coagulates [31,32]. The uncoated liposomes (PLF1–PLF4) showed a negative zeta potential value in comparison to the chitosan-coated liposomes (PLF2C1–PLF3C2). The difference between the uncoated and coated liposomes was statistically significant (*p* < 0.001). The chitosan-coated liposomes showed a marked change in zeta potential value from negative values to positive values. The presence of cationic-charged chitosan is the main reason behind such change. The presence of chitosan on the surface of the liposome indicates high stability due to electrostatic interaction between liposomes and chitosan [33]. Therefore, the coating of liposomes with chitosan changed the particle size and surface charge but did not influence the size distribution (PDI).

### 2.3. Encapsulation Efficiency

The encapsulation efficiency of the prepared uncoated and coated liposomes was calculated to assess the amount of PPN in the liposomes (Table 2). The uncoated liposomes (PLF1–PLF4) showed an encapsulation efficiency in the range of 69.5 ± 1.04% to 79.8 ± 0.03%. The cholesterol and surfactant ratio may be the reason for the variation in the encapsulation efficiency. The higher encapsulation of PPN is due to the lipophilic nature. The increase in the alkyl chain length of the surfactant gives the higher encapsulation. It gives greater lipophilicity to the environment [34]. There was a slight decrease in encapsulation efficiency observed for the chitosan-coated liposomes. The encapsulation efficiency was found to be in the range of 67.7 ± 0.61% to 75.53 ± 1.1%. Chitosan was able to prevent the leakage of encapsulated PPN from liposomes [35]. There was a slight decrease in encapsulation efficiency observed after coating with chitosan. This difference was found to be insignificant.

### 2.4. FTIR Spectroscopy

**Figure 2:**
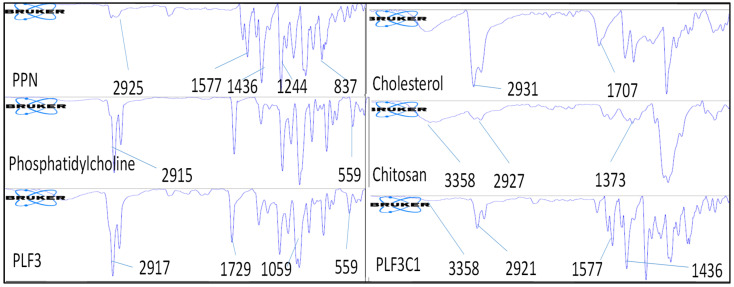
IR spectra of pure piperine, carriers and prepared chitosan-coated (PLF3C1) and uncoated liposomes (PLF3).

The imputed FTIR vibrations of pure PPN, carrier’s chitosan, cholesterol and phosphatidylcholine were compared with the prepared formulations PLF3C1 and PLF3. The characteristic peaks of the functional groups with the frequencies are shown in Figure 2. PPN, a pungent alkaloid, belonging to the “vanilloid family of compounds”, showed a characteristic peak at 2925.24 cm^−1^ (aromatic C-H stretching). The peaks for carbonyl stretching vibrations and C=C ethylenic and aromatic stretching vibrations were exhibited at 1627.32, 1577.19 and 1436.53 cm^−1^, respectively [1]. The C-O-C stretching vibration peaks were observed at 1119.44 cm^−1^ for the alkaloid. Cholesterol exhibited a broad and intense O-H stretching vibration at 3402.57cm^−1^. The distinct peak for CH_2_ symmetric stretching vibration was observed at 2931.70 cm^−1^. The double bond present in the second ring of cholesterol exhibited a frequency at 1707.01 cm^−1^. Chitosan showed a characteristic peak of OH stretching vibration at 3358.26 cm^−1^, whereas C-H peaks and CH_2_-OH vibration peaks were exhibited at 2927.18 and 1373.78 cm^−1^. A small band at 1550 cm^−1^ for N-H bending of amide II was also observed for the excipient. Phosphatidylcholine (PC) depicted an absorbance band at 559.82 cm^−1^, which is assigned to the CN + (CH_3_)_3_ deformation vibration. It also showed an absorption band at 2915.57 cm^−1^, which is attributed to an acid carboxylic group (RCOO^−^). The formulation prepared with chitosan exhibited broad OH stretching vibration peaks at 3358.29 cm^−1^, which are attributed to the excipient chitosan. There was a slight modification in the peaks of tertiary nitrogen, C=C ethylenic and C=C aromatic peaks observed at 2921.86, 1577.95 and 1436.75 cm^−1^ as compared to pure PPN. These peaks were due to the presence of used carriers. In contrast, the formulation PLF3 exhibited a change in its peaks, as compared to pure PPN. PLF exhibited the peaks of CN + (CH_3_)3 at 2917.03 cm^−1^. The given peak was not observed for PLF3C1. No significant shift was found in the wavenumbers of characteristic peaks in the chitosan-coated and uncoated liposomes, indicating the absence of physicochemical interaction and incompatibility for PPN in the liposomes.

### 2.5. Transmission Electron Microscopy

**Figure 3:**
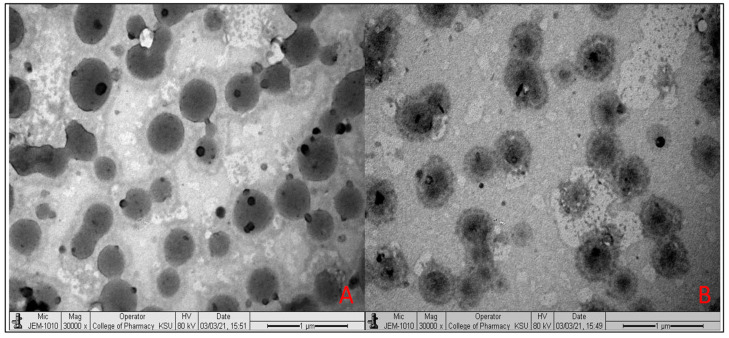
Surface morphology of (**A**). uncoated PPN liposomes (PLF3) and (**B**). chitosan-coated PPN liposomes (PLF3C1).

The surface morphology of the selected PPN liposome (PLF3) and chitosan-coated PPN liposome (PLF3C1) formulations was evaluated by the TEM technique. The image showed nanosized spherical vesicles of PPN liposomes (Figure 3A) with no significant difference in size. The chitosan-coated PPN liposomes (Figure 3B) showed a layer of chitosan over the liposomes. It confirms that the coating of chitosan did not alter the shape of liposomes.

### 2.6. Mucoadhesive Study

The mucoadhesive efficiency of the selected PLF3 and PLF3C1 was assessed by adsorption techniques. The concentration of adsorbed mucin was determined to check the mucoadhesive property of the prepared formulations. The chitosan-coated liposomes showed a 2.8-fold higher mucoadhesive property than the uncoated liposomes. This is attributed to the interaction between cationic-charged chitosan and anionic-charged mucin, hydrophobic interaction and hydrogen bonding [36]. The cationic charge of chitosan also facilitates the permeation of chitosan liposomes across the negatively charged biological membranes. It can interact with negatively charged mucin secreted from intestinal epithelial cells [27]. Thus, it has been concluded that the enhanced mucoadhesive property potentially leads to longer residence time in the GIT region and also gives better therapeutic efficacy [37].

### 2.7. Drug Release

**Figure 4:**
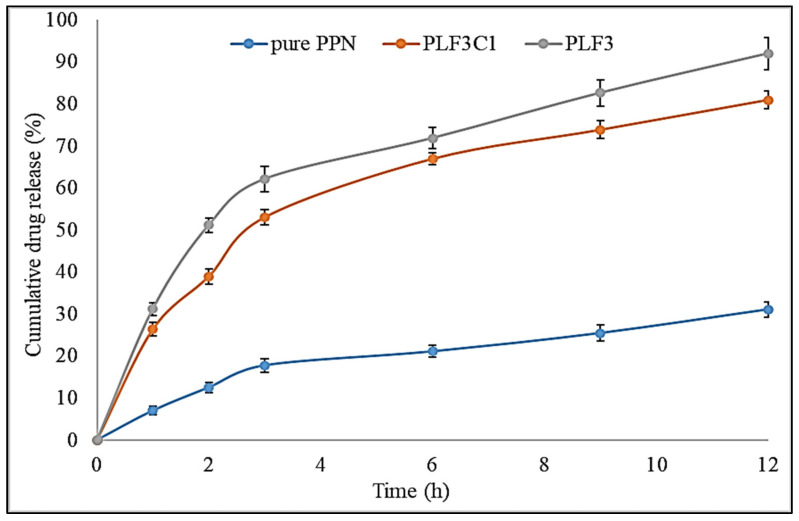
Comparative drug release study profile of pure PPN, PPN liposomes (PLF3) and chitosan-coated PPN liposomes (PLF3C1). The study was performed in triplicate and data are shown as mean ± SD.

The release patterns of the comparative in vitro drug release study are shown in Figure 4. Pure PPN, PLF3C1 and PLF3 showed cumulative drug release of 32.4 ± 2.4%, 81 ± 2.8% and 92 ± 1.6% at 12 h release study, respectively. The release was found to be remarkably high from the prepared formulations. Pure PPN showed poor drug release due to poor solubility and the selected PLF3 and PLF3C1 showed a significantly (*p* < 0.001) higher release. There was an initial fast release for 2 h and after that prolonged release was achieved for the remaining time. The initial fast release was found due to the presence of PPN at the surface of the vesicles and it was immediately available for the release media. The prolonged drug release was found due to the encapsulation of PPN inside the lipid vesicle leading to slow release. In the case of chitosan-coated liposomes, chitosan further delayed the drug release. The drug has to diffuse through both the lipid vesicle and chitosan layer [38]. The slow-release pattern is ideal for prolonged oral delivery systems. The difference in release patterns between uncoated and coated liposomes is attributed to easier disruption of the structural integrity of uncoated than-coated liposomes [39].

### 2.8. Permeation Study

The permeation study was performed for PLF3 and PLF3C1 formulations, and the results were compared with pure PPN. The permeation flux was found to be higher for the prepared PLF3C1 (557.01 ± 6.2 µg/cm^2^/h) and PLF3 (415.1 ± 1.1 µg/cm^2^/h) than pure PPN (153.33 ± 4.04 µg/cm^2^/h). The amount of drug permeation from selected PLF3 and PLF3C1 was found to be significantly higher (*p* < 0.001) than pure PPN. The poor solubility of PPN leads to poor permeability and consequently poor bioavailability. There was a highly significant (*p* < 0.001) enhancement of 2.7-fold and 3.6-fold in the permeation flux achieved in PLF3 and PLF3C1 than pure PPN, respectively. The permeation flux of PLF3 and PLF3C1 was also compared, and the result showed a significant enhancement of 1.34-fold (*p* < 0.001) from PLF3C1. The findings of this study are in agreement with the previously published literature [30,37]. The reason for the higher permeation from liposomes (PLF3) may be due to the presence of surfactant in the liposomes, which leads to enhancement in the solubility of poorly soluble drugs. The presence of cholesterol and lipids also helps reduce the barrier property at the site of absorption [37]. In the case of PLF3C1, the presence of a chitosan coat showed an enhanced mucoadhesive property inducing disruption of the tight junction in the mucosa. The positive charge of the chitosan amino group interacts with the negative charge of sialic acid of the intestinal membrane. Thus, it gives greater permeation across the mucosal surface [40]. This observation also showed that encapsulated PPN in chitosan-coated liposomes is not available for the P-gp pump and can be easily transported across the intestinal wall for absorption [41].

### 2.9. Antioxidant Activity

**Figure 5:**
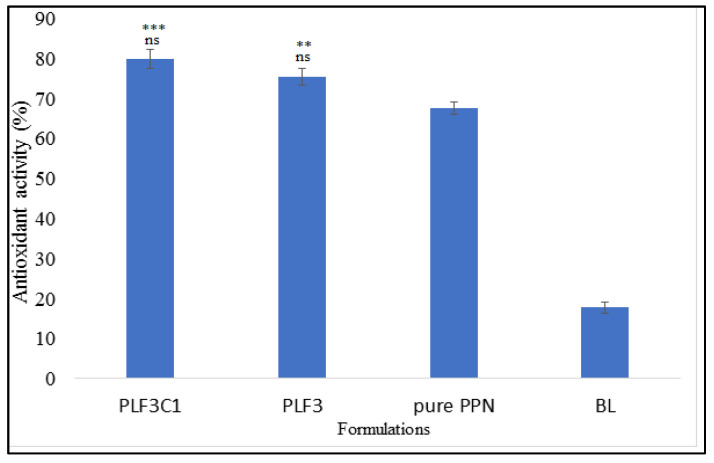
Antioxidant activity of the tested samples using the DPPH method. The Tukey–Kramer multiple comparison test was utilized to analyze the statistically significant difference between each group. Difference was considered significant if *p* < 0.05. ns = not significant when compared between PPN liposomes (PLF3) and chitosan-coated PPN liposomes (PLF3C1); *** = *p* < 0.001 when compared with pure PPN; ** = *p* < 0.01 when compared with pure PPN.

Antioxidant activity plays a major role in the biological activity of plant-based bioactive compounds. Therefore, it is necessary to evaluate that the used excipients do not interfere with the antioxidant property of the compounds. Antioxidant activity was performed by the DPPH assay method. There was a distinct difference in the activity of all tested groups, as shown in Figure 5. The selected formulations PLF3 (*p* < 0.01) and PLF3C1 (*p* < 0.001) showed a significantly higher antioxidant property than pure PPN. The presence of used excipients in the formulations enhances the antioxidant activity of pure PPN. PLF3C1 showed slightly higher activity at the tested concentration than PLF3. Higher activity was observed due to the enhanced solubility of pure PPN. There was a nonsignificant difference observed between PLF3 and PLF3C1. From the activity, it was concluded that the DPPH radical scavenging activity of PPN was enhanced after encapsulation into liposomes and chitosan-coated liposomes. Similar findings are reported in the published literature [42]. There was weak activity also observed for blank liposomes due to the presence of phosphatidylcholine [43].

### 2.10. Cell Viability

**Figure 6:**
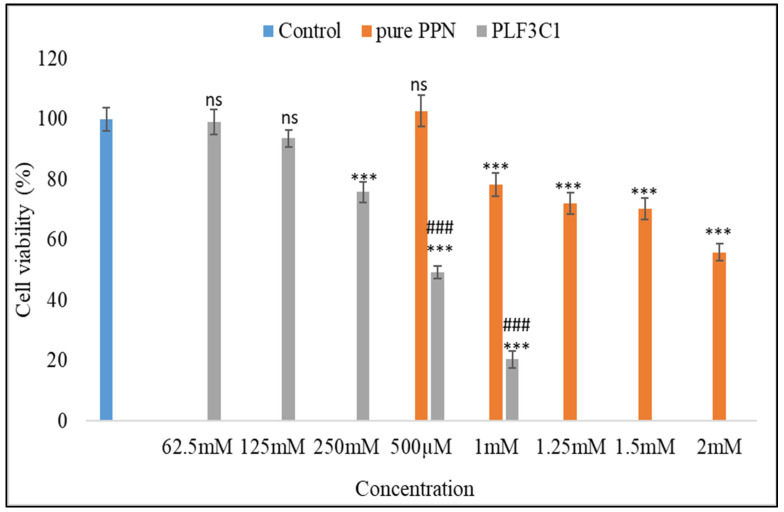
Effect of different concentrations of pure PPN and LF3C1 on the viability of MCF7 cells evaluated by MTT assay. Data are presented in percent (%) in comparison to control as 100%. The Tukey–Kramer multiple comparison test was utilized to analyze the statistically significant difference between different concentration exposures and control. Difference was considered significant if *p* < 0.05. ns = not significant when compared with control; *** = *p* < 0.001 when compared with control; ^###^ = *p* < 0.001 when compared with the same concentration groups of piperine standard.

Data reveal that lower concentrations of both pure PPN and PPN liposome formulations have minimal effects on the cell viability of MCF7, which is reduced with increasing concentration (Figure 6). Cell viability percentage for different pure PPN concentrations was 102.78 ± 4.94 (500 µM,), 78.28 ± 4.75 (1 mM, *p* < 0.001), 72.06 ± 2.03 (1.25 mM, *p* < 0.001), 70.25 ± 2.4 (1.5 mM, *p* < 0.001) and 55.95 ± 4.66 (2 mM, *p* < 0.001). Cell viability percentage for different concentrations of PPN formulation was 99.03 ± 2.75 (62.5 µM), 93.69 ± 5.04 (125 µM), 75.91 ± 2.55 (250 µM, *p* < 0.001), 49.2 ± 3.18 (500 µM, *p* < 0.001) and 20.46 ± 1.75 (1 mM, *p* < 0.001). When comparing the same concentration groups in both exposures (500 µM and 1 mM), they were found to be significantly different (*p* < 0.001). When comparing the effects, it clearly appears that the formulation of PPN has enhanced growth inhibitory effects in MCF7 cells at lower concentrations in comparison to pure PPN. Figure 6 shows these effects and the comparison. In the present investigation, the IC_50_ of pure PPN in MCF7 cells was found to be 2.12 mM, reduced by the formulation to 595.98 µM, which is 3.55 times lower than pure PPN. In the cell viability assay, or MTT assay, it is revealed that PPN has inhibitory effects on the growth of MCF7 cells, which is found to be concentration dependent. However, this may also be time dependent, but the present investigation did not require a time-dependent investigation using the cell viability assay. Data from the cell viability assay also highlighted that the formulation containing PPN significantly enhanced these effects in terms of reducing the IC_50_ when compared with standard PPN.

## 3. Materials and Methods

### 3.1. Materials

Piperine (PPN) was purchased from Beijing Mesochem Technology Co. Pvt Ltd. (Beijing, China). Cholesterol and Phospholipon^®^ 90H (phosphatidylcholine (PC)) were purchased from Alpha Chemika and Lipoid (Lipoid GMBH). Sodium cholate was procured from Sigma-Aldrich (Merck), Mumbai, India. Chitosan (degree of deacetylation: >95%) was purchased from UFC Biotechnology, Amherst, NY, USA.

### 3.2. Formulation of Liposomes

Piperine-loaded liposomes were prepared by the thin-layer dispersion method using a rotary evaporator [44]. The weighed quantities of cholesterol, sodium cholate and phosphatidylcholine were used in this study, and the compositions are shown in Table 1. Each ingredient was added in a methanol chloroform mixture (6 mL; 1:1) and shaken until complete solubilization in a round-bottom flask. PPN was added to the above solution. The organic solvent was then evaporated using a rotary evaporator to form a thin lipid film. The flask was kept in a desiccator overnight to remove the residue of organic solvents. The lipid film was hydrated with phosphate buffer (pH 7.4) for 60 min to remove the thin film completely. The prepared samples were collected and further coated with chitosan (0.2% and 0.4% *w*/*v*) to enhance the mucoadhesive property. Chitosan solution (0.2% and 0.4% *w*/*v*) was prepared in acetic acid and added dropwise to the prepared liposome formulation with magnetic stirring. The sample was stirred for 30 min for the complete coating of liposomes. The uncoated and coated liposome formulations were further characterized for different parameters.

### 3.3. Vesicle Characterization

The vesicle characterization (size, PDI and zeta potential) of the uncoated PPN liposomes and chitosan-coated PPN liposomes was performed by Malvern zetasizer. The samples (0.1 mL) were taken and diluted 100-fold to avoid the multiscattering of the vesicles. The samples were transferred into a plastic cuvette to measure the size and PDI at 25 °C. The study was performed in triplicate.

### 3.4. Encapsulation Efficiency

The encapsulation efficiency of the prepared uncoated PPN liposomes and chitosan-coated PPN liposomes was performed by an indirect method. Each sample (1.25 mL) was taken in the tube and centrifuged at 4000 rpm for 30 min. The supernatant (0.5 mL) was separated and diluted with phosphate buffer at pH 7.4. PPN content in each sample was evaluated by a UV spectrophotometer at 343 nm. The amount of encapsulated PPN was calculated from the below equation:

(1) $$\%\mathrm{EE}=\frac{\left(\mathrm{Total}\;\mathrm{PPN}-\mathrm{Free}\;\mathrm{PPN}\right)}{\mathrm{Total}\;\mathrm{PPN}}\times 100$$

### 3.5. Fourier-Transform Infrared Spectroscopy (FTIR)

An FTIR spectroscopy study was performed to evaluate the interaction between pure PPN and excipients in the formulations. The evaluation was performed by evaluating the changes in peak shape, position and intensity. The spectra of pure PPN, cholesterol, phosphatidylcholine, chitosan and their formulations PLF3 and PLF3C1 were used to evaluate the interaction. The spectrophotometer (ATR-FTIR, Bruker, Alpha, Ettlingen, Germany) was used to study the samples.

### 3.6. Surface Morphology

The surface morphology of PLF3 and PLF3C1 was evaluated by a transmission electron microscope (TEM: JEM-1010, Tokyo, Japan). The prepared samples were diluted with ultrapure water, and later, one drop of each sample was carefully taken on a clean copper grid and air-dried. The sample was then stained with phosphotungstic acid (2%) and air-dried. Finally, the grid was visualized under a high-resolution microscope to check the morphology.

### 3.7. Drug Release

The comparative study was evaluated for the prepared PPN-uncoated and chitosan-coated liposomes. The samples (~15 mg PPN) were filled in a dialysis bag membrane (molecular weight cutoff: 12,000 KDa) and dipped in the release media (phosphate buffer, pH 7.4). The release media (900 mL) was used for the study, the release content (5 mL) collected at the 0, 1, 2, 3, 6, 9 and 12 h time points and replaced with fresh release media. The collected samples at each time point were evaluated by a UV spectrophotometer (Shimadzu 1601 PC, Japan) at 343 nm. The absorbance was noted. and drug release was calculated from the standard plot. The drug release study was compared with the same amount of pure PPN to check the difference in release pattern.

### 3.8. Mucoadhesive Efficiency

The mucoadhesive efficiency (ME) study of PLF3 and PLF3C1 was performed by the adsorption method using a mucin concentration [37]. The samples were mixed with the same volume (1:1) of mucin solution (1 mg/mL). Samples were then incubated at 37 °C for 2 h and centrifuged for 1 h at 5000 rpm. The supernatant was collected, and mucin content was estimated spectrophotometrically with an appropriate dilution. The amount of mucin adsorbed was assessed by taking the difference between the amount of mucin added and free mucin. The mucin content was calculated using the formula:

$$\mathrm{ME}=\left[\frac{\mathrm{Ci}-\mathrm{Cf}}{\mathrm{Ci}}\right]\times 100$$

where, Ci is the initial mucin concentration, and Cf is the final mucin concentration.

### 3.9. Permeation Study

The study was performed using a diffusion cell having an effective area of 1.5 cm^2^ and a volume of 20 mL. The selected formulations of PLF3, PLF3C1 and pure PPN (~2 mg) were filled in the donor cell. The diffusion media was filled in the receptor cell, and the rat intestine was fixed between the receptor and donor cell. The temperature was maintained at 37 °C with continuous stirring. The selected formulations were filled in the donor cell, and diffused content was collected from the receptor cell filled with phosphate-buffered saline. After a specific time (0.5, 1, 2, 3, 4, 6 h), a 1 mL sample was taken and replaced with fresh media. The released content was filtered and diluted further to estimate the amount of drug permeated using a spectrophotometer.

### 3.10. In Vitro Antioxidant Study

The antioxidant activity was performed using the DPPH radical scavenging method [45]. The selected formulations of PLF3, PLF3C1 and pure PPN were prepared in a stock solution of 1 mg/mL. The samples were further diluted to make a concentration of 50 µg/mL. Separately, methanolic DPPH solution was prepared in a concentration of 25 μM. A 0.5 mL volume of the test samples was mixed with 3 mL of the methanolic DPPH solution and left for 30 min in the dark to complete the reaction. The absorbance was measured at 517 nm by a spectrophotometer. The extent of change in color from violet to colorless quantified as a decrease in absorbance depends on the antioxidant property of the samples. The antioxidant property of each sample was calculated by using the formula:

$$\mathrm{Antioxidant}\;\mathrm{activity}=\left(\frac{\mathrm{As}-\mathrm{At}}{\mathrm{As}}\right)\times 100$$

where, As is the absorbance of DPPH, and At is the absorbance of the test.

### 3.11. In Vitro Cell Line Study

Cell Viability Assay

The effect of different concentrations of pure PPN and PLF3C1 on MCF7 cell viability was assessed by using MTT (3-(4,5-dimethylthiazol-2-yl)-2,5-diphenyl tetrazolium bromide). Briefly, MCF7 cells were seeded into a 96-well plate, around 15,000 cells per well in a 100 µL cell media (DMEM, 10% FBS). Seeded cells were incubated overnight at 37 °C and 5% CO_2_ to allow proper adherence. Different concentrations of pure PPN and the formulations were added in different wells to see their cytotoxic effects. DMSO was used to make stock solutions of the standard and the formulation that were serially diluted in a 96-well plate using serum-free media. The final concentration of the DMSO was kept below 1% to avoid any undesired effects on the cells. The same DMSO diluted in the cell culture media was used as vehicle control. After several pilot experiments, a range of concentration was selected for pure PPN (500 µM, 1 mM, 1.25 mM, 1.5 mM and 2 mM) and PLF3C1 (62.5 µM, 125 µM, 250 µM, 500 µM and 1 mM). The assay was conducted in quadruplicate. After 24 h of exposure to PPN, 10 µL of MTT solution (5 mg/mL PBS) was added to each well except the blank. The cells were incubated for 4 h to allow the metabolism of MTT by viable cells. Cell media was removed from all of the wells and 100 µL of DMSO was added to dissolve the formazan of MTT. After 30 min of incubation, the plate was read at 570 nm using DMSO as blank.

### 3.12. Statistical Analysis

Data are presented as mean ± SD. The raw data were analyzed using GraphPad InStat demo version (GraphPad Software Inc., La Jolla, CA, USA). Data were subjected to One-way ANOVA, followed by the Tukey–Kramer multiple comparison test to analyze statistically significant different concentration exposures and control.

## 4. Conclusions

Chitosan-coated liposomes were prepared to enhance the in vitro therapeutic efficacy of PPN, a water-insoluble drug. PPN-loaded liposomes were prepared by the thin-film hydration method and further coated with mucoadhesive polymer chitosan. The prepared PPN liposomes and chitosan-coated PPN liposomes were evaluated for different physicochemical parameters. The formulations showed nanometric size, low PDI, optimum zeta potential and high encapsulation efficiency. The enhanced PPN release and permeation was achieved with the prepared formulations. The chitosan-coated liposomes showed a high mucoadhesive property and comparable antioxidant effect than pure PPN. Furthermore, chitosan-coated liposomes showed enhanced effects in terms of reducing the IC_50_ in MCF7 cells when compared with pure PPN.

## Figures and Tables

**Table 1:** Composition of piperine liposome and chitosan coated piperine liposomes.

Formulations	Phosphatidyl Choline (%)	Sodium Cholate (%)	Cholesterol (%)	Chitosan (%)
PLF1	85	10	5	-
PLF2	80	15	5	-
PLF3	75	20	5	-
PLF4	70	25	5	-
PLF2C1				0.2
PLF2C2				0.4
PLF3C1				0.2
PLF3C2				0.4

**Table 2:** Physicochemical characterization of piperine-loaded uncoated and chitosan-coated liposomes. The study was performed in triplicate (*n* = 3), and data are shown as mean ± SD.

Formulations	Size (nm)	PDI	Zeta Potential (mV)	Encapsulation Efficiency (%)	Drug Release (%)
PLF1	213.1 ± 6.3	0.27	−14.3 ± 2.07	69.5 ± 1.04	77.0 ± 1.2
PLF2	204.2 ± 8.1	0.23	−8.5 ± 1.07	77.01 ± 1.42	88.3 ± 0.9
PLF3	178.1 ± 4.5	0.19	−7.1 ± 0.41	79.8 ± 0.03	92.0 ± 1.3
PLF4	165.7 ± 7.4	0.29	−8.2 ± 0.47	71.4 ± 1.35	86.9 ± 1.5
PLF2C1	243.4 ± 7.5	0.28	20.3 ± 0.92	71.1 ± 1.54	79.3 ± 1.7
PLF2C2	234.5 ± 4.3	0.26	24.1 ± 0.65	67.7 ± 0.61	73.1 ± 1.2
PLF3C1	211.6 ± 9.8	0.24	25.4 ± 0.66	74.9 ± 1.24	80.9 ± 2.2
PLF3C2	223.8 ± 2.7	0.29	29.8 ± 0.77	75.53 ± 1.1	75.7 ± 1.1

## Sample Availability

Samples of the compounds are not available from the authors.
